# Activity Patterns of Eurasian Lynx Are Modulated by Light Regime and Individual Traits over a Wide Latitudinal Range

**DOI:** 10.1371/journal.pone.0114143

**Published:** 2014-12-17

**Authors:** Marco Heurich, Anton Hilger, Helmut Küchenhoff, Henrik Andrén, Luděk Bufka, Miha Krofel, Jenny Mattisson, John Odden, Jens Persson, Geir R. Rauset, Krzysztof Schmidt, John D. C. Linnell

**Affiliations:** 1 Bavarian Forest National Park, Department of Conservation and Research, Grafenau, Germany; 2 Chair of Wildlife Ecology and Management, Albert Ludwig University of Freiburg, Freiburg, Germany; 3 Ludwig Maximilian University Munich, Statistical Consulting Unit, Department of Statistics, Munich, Germany; 4 Grimsö Wildlife Research Station, Department of Ecology, Swedish University of Agricultural Sciences, Riddarhyttan, Sweden; 5 Department of Research and Nature Protection, Šumava National Park Administration, Kašperské Hory, Czech Republic; 6 University of Ljubljana, Biotechnical Faculty, Ljubljana, Slovenia; 7 Norwegian Institute for Nature Research, Trondheim, Norway; 8 Mammal Research Institute, Polish Academy of Sciences, Białowieża, Poland; University of Texas Southwestern Medical Center, United States of America

## Abstract

The activity patterns of most terrestrial animals are regarded as being primarily influenced by light, although other factors, such as sexual cycle and climatic conditions, can modify the underlying patterns. However, most activity studies have been limited to a single study area, which in turn limit the variability of light conditions and other factors. Here we considered a range of variables that might potentially influence the activity of a large carnivore, the Eurasian lynx, in a network of studies conducted with identical methodology in different areas spanning latitudes from 49°7′N in central Europe to 70°00′N in northern Scandinavia. The variables considered both light conditions, ranging from a day with a complete day–night cycle to polar night and polar day, as well as individual traits of the animals. We analysed activity data of 38 individual free-ranging lynx equipped with GPS-collars with acceleration sensors, covering more than 11,000 lynx days. Mixed linear additive models revealed that the lynx activity level was not influenced by the daily daylight duration and the activity pattern was bimodal, even during polar night and polar day. The duration of the active phase of the activity cycle varied with the widening and narrowing of the photoperiod. Activity varied significantly with moonlight. Among adults, males were more active than females, and subadult lynx were more active than adults. In polar regions, the amplitude of the lynx daily activity pattern was low, likely as a result of the polycyclic activity pattern of their main prey, reindeer. At lower latitudes, the basic lynx activity pattern peaked during twilight, corresponding to the crepuscular activity pattern of the main prey, roe deer. Our results indicated that the basic activity of lynx is independent of light conditions, but is modified by both individual traits and the activity pattern of the locally most important prey.

## Introduction

The sequence of day and night is the dominant “Zeitgeber”, which synchronizes the internal clocks of most terrestrial organisms [1]. This functionality enables the organisms to proactively adapt their biochemical and physiological processes and their behaviour to the 24-h environment [2]. This general circadian pattern of behaviour can be modified by changing environmental conditions, such as season [3] and weather [4], or by social [5] and individual traits [6]. Also the avoidance of predators [7] and humans [8], [9] or patterns of food availability [10] can influence the activity of animals.

The circadian rhythm of felids is generally considered to be crepuscular or nocturnal [11], although the anatomical structure of their eyes makes them well suited to function under a wide range of light conditions [12]. The daily predatory activity of felids with large geographic ranges varies from fully nocturnal to circadian and seems to be flexible both among and within species [13]. Important factors driving felid activity rhythms seem to be the activity of their main prey [14]–[16], and nocturnal activity is a primitive form of antipredator behaviour that has developed in eutherian mammals during the Mesozoic Era (Nocturnal Bottleneck Hypothesis) to which predators have to respond [17]. For instance, the activity of jaguars (*Panthera onca*) and pumas (*Puma concolor*) coincides with the nocturnal activity of armadillos (*Dasypus novemcintus*) and pacas (*Aguti paca*) [18]. Similarly, pumas and Andean cats (*Leopardus jacobita*) in the High Andes follow the activity of mountain vizcachas (*Lagidium viscacia*) [7]. Also lions (*Panthera leo*) take advantage of prey vulnerability, stalking at night in plains with little cover and diurnally in forests when prey congregates along rivers [19].

Previous studies of Eurasian lynx have shown that the species has a nocturnal or crepuscular rhythm of activity, which has been shown to vary markedly with temperature, sex, reproductive status and prey behaviour [14], [15], [20], [21]. The activity of reproducing females is less nocturnal than that of adult males, although the duration of total daily activity of both sexes is similar [14]. The general pattern of activity of both males and females does not change over the year, but males are active longer during the mating season as they also travel longer distances and cover larger ranges, and females travel less when they have small kittens [14], [15], [22], [23].

Most studies of carnivore activity patterns, including studies of lynx, have been conducted within single study sites or geographically limited regions with a relatively constant day–night cycle. But at high or low latitudes, where the sun remains constantly above the horizon (polar day) or below the horizon (polar night) during the peak of summer and winter, a constant periodicity seems to be of no advantage [24]. van Oort et al. [25] found that reindeer (*Rangifer tarandus*) on the high arctic islands of Svalbard, with associated polar nights and polar days, do not exhibit the 24-h locomotor periodicity observed in central Norway, which has a day–night cycle. Instead, reindeer activity is polycyclic and determined by the rumination cycle. The authors conclude that species have no selective advantage in maintaining daily cycles in a non-rhythmic environment. They further hypothesized that in the absence of a circadian rhythmicity, the activity of herbivorous animals is controlled by their feeding cycles, and as a consequence, also the activity patterns of predators preying on reindeer should adapt during polar night and polar day.

The light intensity between new moon and full moon ranges over three orders of magnitude [26]. Moonlight therefore may influence the activity of nocturnal carnivores that detect their prey visually, i.e. when light intensity increases, the predator should be able to detect and catch prey more easily. On the other hand, moonlight might reduce predation risk if the predators need darkness to successfully attack visually-oriented prey [27]. Only a few empirical studies have assessed the role of the moon phase on the activity of felid predators and their prey [18], [27], [28]. These studies indicate that the response of prey species to moonlight also influences the activity of the predator. For example, moon phase has no overall positive influence on jaguar activity levels, but when armadillos, their most important prey, avoided foraging above ground during moonlight nights, jaguar activity declined [18]. The activity of bobcats (*Lynx rufus*) was lower on nights with a new moon and higher during full moon; this pattern was related to the activity of their prey species, mostly small mammals, which move and forage less during high lunar illumination to reduce predation risk [28]. Because no study has indicated an influence of moonlight on the activity of the lynx prey species red deer, roe deer and reindeer and because lynx is an efficient stalking predator that can use any hiding cover to approach its prey, lynx are believed to hunt successfully with or without illumination by moonlight.

In this study, we analysed the activity patterns of Eurasian lynx along a >20 degree latitudinal range with major differences in light conditions, ranging from areas in the south that always have a complete day–night cycle to areas in the north with polar night and polar day. Our objective was to use this immense degree of variation in light cycle to disentangle the relative impacts of different external and internal factors on lynx activity. Based on what is known about carnivore activity patterns, we tested the following predictions: (1) the general activity level is not influenced by daylight duration, (2) the animals exhibit a crepuscular behaviour during the time of year with a full day–night cycle, (3) the animals show distinct circadian patterns also during polar day and polar night, (4) lynx activity remains constant with increasing intensity of moonlight, and (5) lynx activity will vary with individual traits, such as sex and age, as reported in previous studies of one locality, regardless of light conditions at different latitudes.

## Materials and Methods

### Data collection and study sites

**Figure 1:**
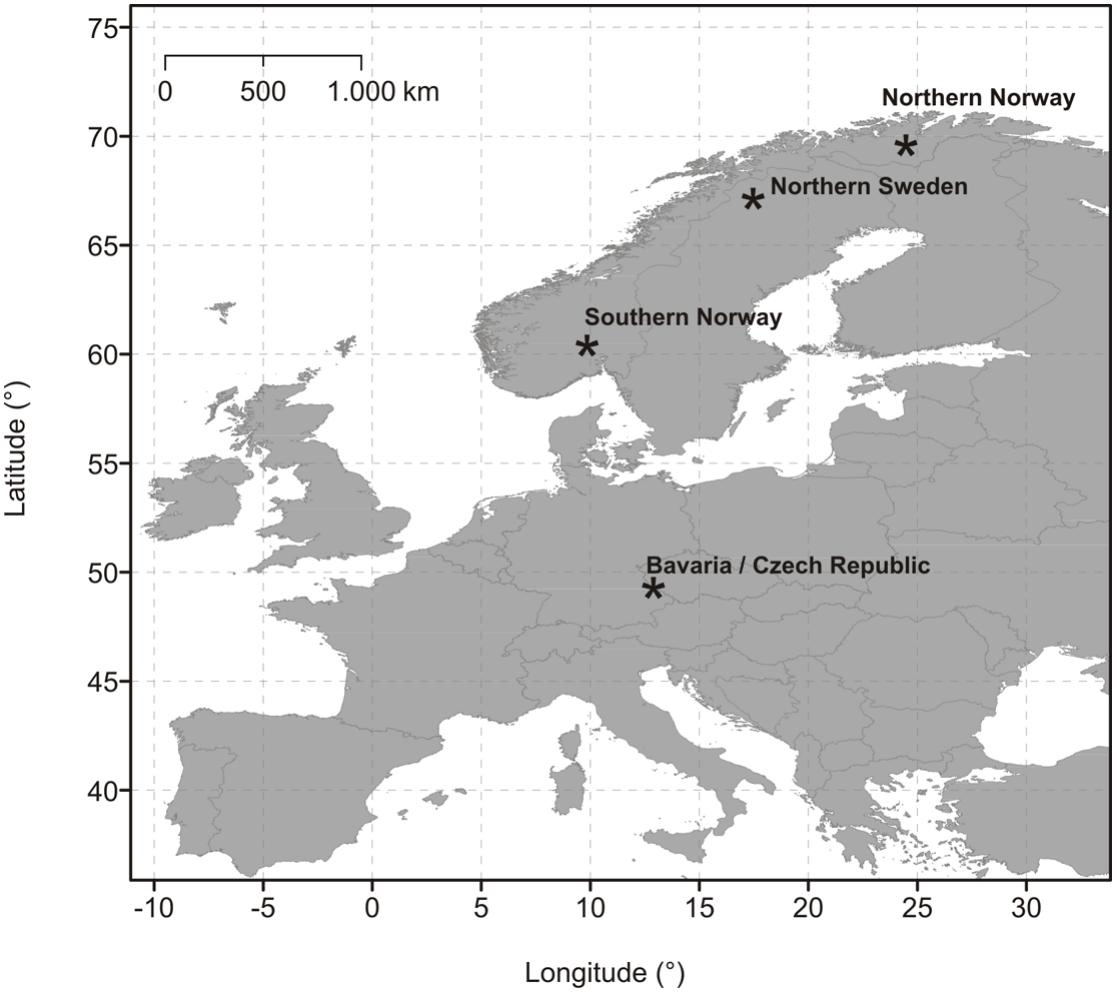
Map of Western Europe with the locations of the four study sites. (See S1 Table “General characteristics of the study sites”, for a detailed description).

We analysed activity patterns of 38 individual Eurasian lynx, using data from four study sites distributed over a latitudinal range of 20 degrees from central Europe to northern Scandinavia (Fig. 1). Detailed information about the study sites can be found in S1 Table and S2 Table.

Lynx were captured following established protocols using a range of methods, including foot snares placed at fresh kills, box traps, darting from a helicopter, and chasing lynx into trees with dogs (Sweden and Norway: [29], [30]; Bavaria, Germany/Czech Republic: [31]). The captured lynx were equipped with GPS collars with either GSM or VHF/UHF download (GPS plus mini, Vectronic Aerospace GmbH, Berlin, Germany). Individuals in Sarek, northern Sweden, were additionally equipped with intraperitoneally implanted VHF transmitters (IMP/400/L, Telonics Inc., Mesa, AZ, USA) for long-term monitoring. When possible, lynx were recaptured to replace the collar when the battery was depleted. The handling protocol has been approved by the Swedish Animal Ethics Committee, the Norwegian Experimental Animal Ethics Committee, and the Ethics Committee of the Government of Upper Bavaria and fulfils their ethical requirements for research on wild animals. In addition, permits for wild animal capture were obtained from the Swedish Environmental Protection Agency, the Norwegian Environmental Agency, and the Government of Lower Bavaria.

The collars also carried an analogue dual-axis accelerometer, which has a dynamic range from −2G to +2G. One axis (axis x) measures acceleration in forward/backward motion, and the other axis (axis y) measures sideward and rotary motion. Changes in static acceleration (gravity) and dynamic acceleration (collar) were measured every 8^th^ second and averaged for 5-min intervals. The value scale ranged from 0 (no activity) to 255 (−2G/+2G). Since the x- and y-activity data are highly correlated [32], [33], we only analysed the x-activity data. We monitored the lynx during periods of different length (min. 62 days, max. 777 days, mean 289 days). We acquired a total of 3,291,947 data points of activity at 5-min intervals for a total of 11,186 monitoring days (Table 1). We only used collars with the same type of activity sensors and firmware, as specified by the manufacturer.

**Table 1:** Distribution of studied lynx according to study site and status and description of the available data.

Area	Numberof lynx	Sex(m/f)	Age (years)(subadult/adult)	Observations(5 min)	Observations(60 min)	Observations(days)
Bavaria-Czech Republic	8	5/3	1/7	666,985	54,840	2,272
Southern Norway	7	4/3	3/4	568,848	47,064	1,950
Northern Sweden	11	3/8	1/10	1,059,765	86,904	3,615
Northern Norway	12	6/6	2/10	996,349	80,568	3,349
Sum	38	18/20	7/31	3,291,947	269,376	11,186

The activity sensors in the collars were sensitive enough to register all movement including head shaking. Based on a previous validation study, we determined that acceleration sensor values from 0 to 27 indicated inactive behaviour [15]. Therefore, we considered the values above 27 as “active”, which likely includes all types of activity (e.g. walking, running, hunting, and interacting). For the analyses, the portion of time (0–100%) the lynx spent active (values>27) in 60-min intervals (activity per hour) was aggregated, as was activity during different time phases of the day, e.g. twilight and night (activity per day phase). In the following, the term activity refers to the percentage of time the lynx were active (values >27).

We obtained times of sunrise/sunset and nautical twilight (centre of the sun is geometrically 12 degrees below the horizon) from the US Naval Observatory using the Naval Oceanography Portal (http://www.usno.navy.mil).

The data which forms the basis for the analysis can be found in S1 Dataset.

### Data analysis

To visualize the activity patterns, we first displayed the acceleration data as an actogram for each study site separately. Actograms illustrate the fine-scale activity pattern of individuals in more detail than plots showing aggregated values for all animals, and are a good demonstration of either variability or constancy of pattern [34]. Then we plotted monthly 24-h patterns of activity for the different seasons and study sites, averaging the portion of time active according to study site and months of the year. All activity data is presented as the portion of time active (0–100%). For example, an activity of 0.4 means that the lynx was active 40% of the time (i.e. with acceleration sensor values >27).

We estimated a linear mixed additive model [35], [36] with the response variable percentage of time the lynx spent active per hour:
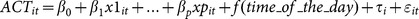




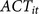
 denotes the activity of animal i at time t, while 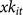 denotes the values of explanatory variables listed in Table 2. The term 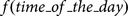 denotes a flexible smooth function of the time of the day, while 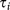 denotes a random intercept for animal I, and 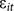 is the error term. 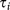 was included since we had repeated measurements of the 38 individuals. The autocorrelation of the residuals from the linear mixed model was 0.294 (lag 1) and 0.104 (lag 2). This is an indication that the chosen models without auto-covariance terms are acceptable. We used the p-splines to estimate the additive component. To verify the results of the models, a sensitivity analysis was performed with a gamma generalized linear mixed model. The results of these fittings were comparable; therefore, the linear mixed model was deemed sufficient.

**Table 2:** Explanatory variables considered in the modelling approach.

Variable	Scale	Value range	Description
Individualanimal	Categorical	(1;38)	Lynx individual id
Daylightduration	Continuous	(0;24)	Length of the day from sunset to sunrise
Moon phase	Continuous	(0;15)	0 = new moon, 15 = full moon
Age	Categorical	Subadult; adult	Adult >1.5 years
Status	Categorical	Male, female,female with kittens	Sex of the lynx and kittens confirmed
Season	Categorical	Spring, summer,autumn, winter	Time of the year according to astronomical definition (21.3; 21.6; 23.9; 21.12)
Study site	Categorical	Bavaria-Czech Republic,southern Norway, northernSweden, northern Norway	Study site

To check whether the activity patterns were dependent on general light conditions, we fitted our model of activity to a data subset that compiled observations during polar night (day on which the sun is below the horizon for 24 h), polar day (day on which the sun is above the horizon for 24 h) and equinox (daylight duration between 11∶00 and 13∶00).

In the second analysis, we fitted models with the daily activity in different day phases. We used the following outcome variables: activity per day (24 h), activity during daylight, activity during twilight morning, activity during twilight evening and activity during the night. The covariates in Table 2, but not the day phase and daylight duration as a quadratic term, were included in these linear mixed models. Note that these models include daily data.

The statistical significance level was set to 5%. The significance levels (p-values) were estimated with a likelihood ratio test for the categorical covariables (e.g. gender, season, age and region) and with a Wald test for all other non-categorical covariables. For the statistical computing, we used the software R (version 2.14.0) [37] and the library “nlme” and “mgcv”.

## Results

### Descriptive analysis

**Figure 2:**
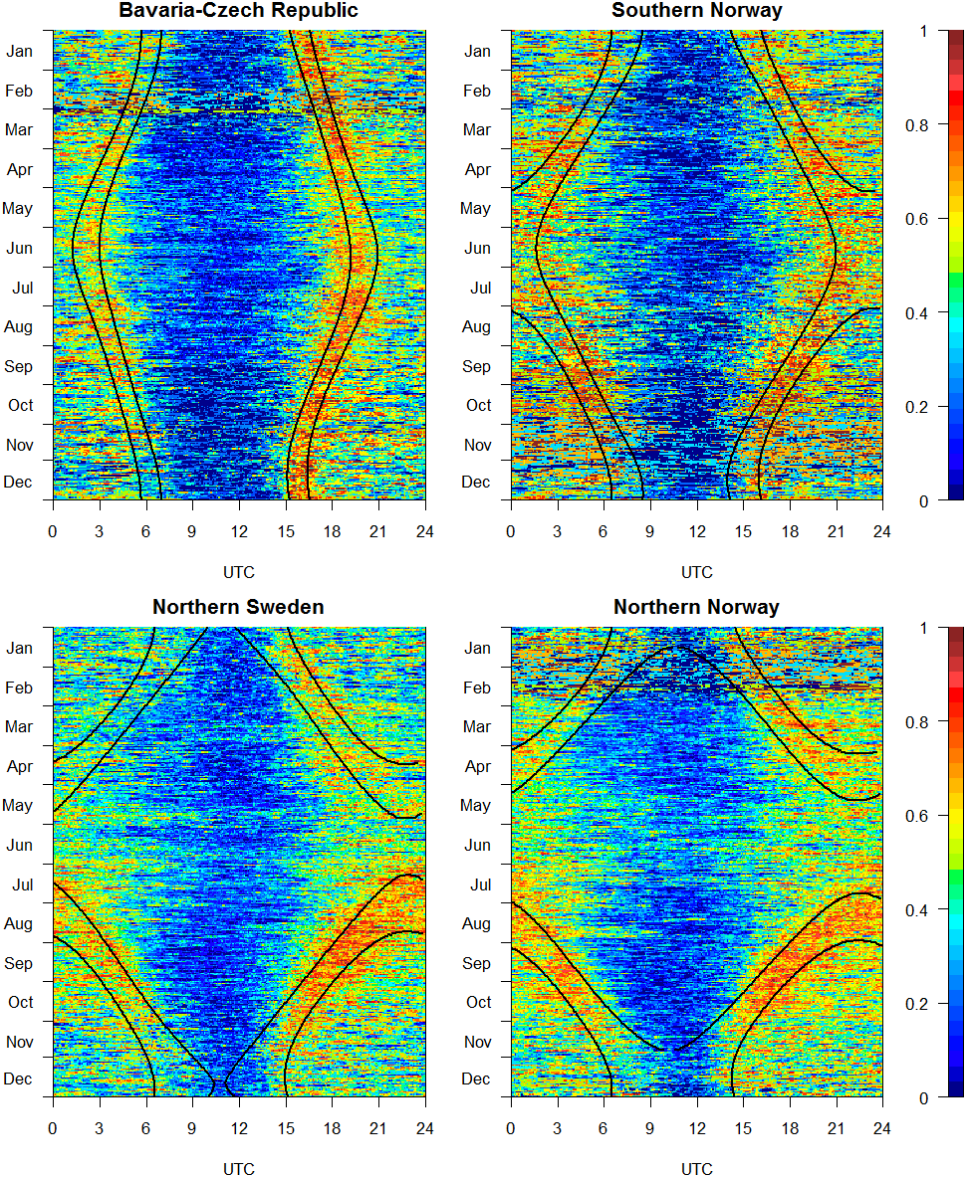
Actograms of Eurasian lynx activity at the four study sites. Activity was measured as forwards-backwards acceleration on a relative scale from 0 to 255. The x-axes indicate the hours of the day (from 0 to 24 UTC; coordinated universal time). The y-axes indicate the days of the year, with each horizontal line representing one day, from January 1 (top line) to December 31 (bottom line). Each coloured pixel corresponds to one mean activity value at one time interval of 5 min averaged over the number of lynx in the corresponding region, ranging from dark blue for little or no activity to red for high activity. Curved black lines from left to right indicate beginning of nautical twilight, sunrise, sunset, and end of nautical twilight.

**Figure 3:**
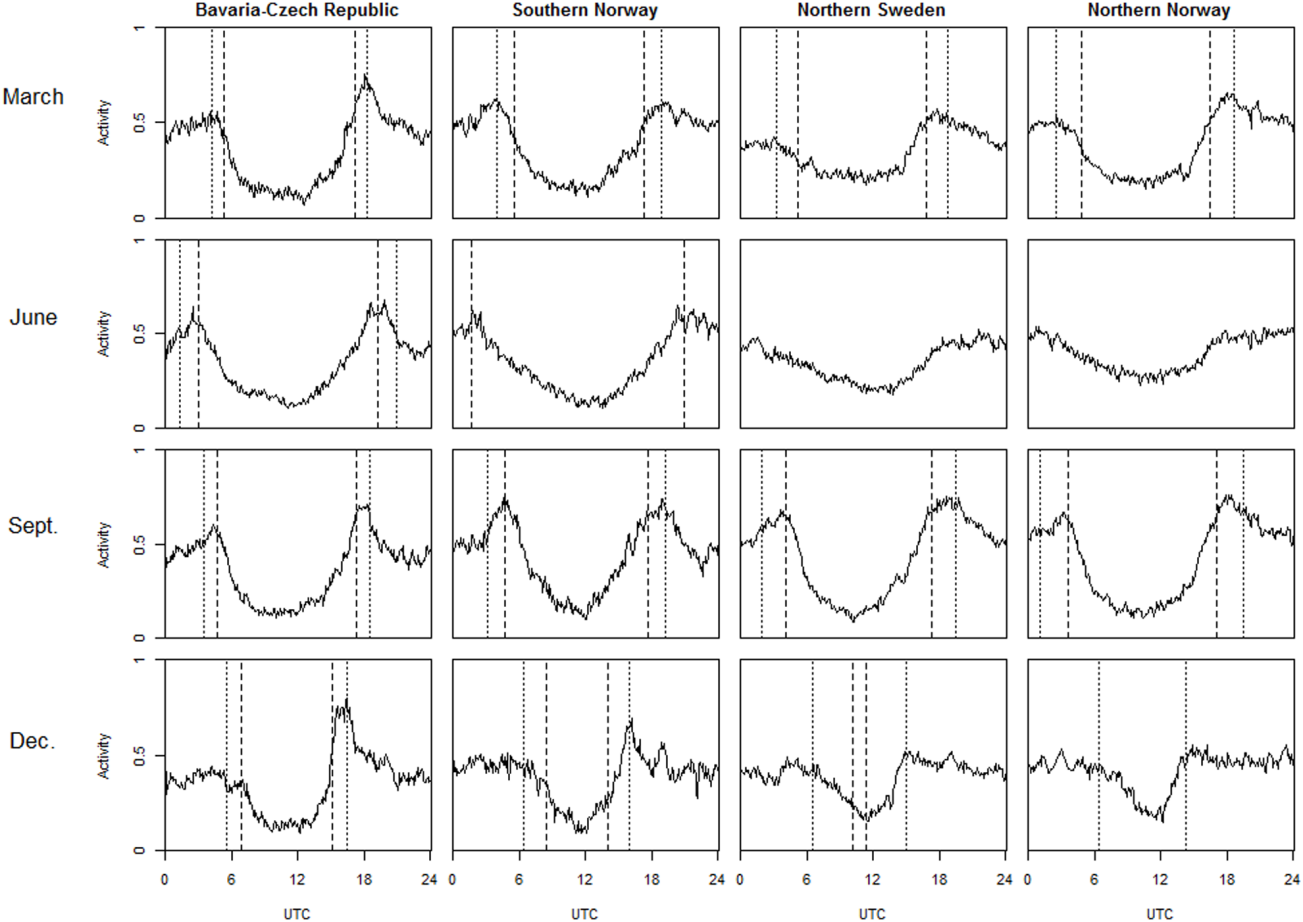
Eurasian lynx daily activity at the four study sites. Each plot shows the portion of time spent active per hour (activity) over the course of a day in the month and averaged over the number of lynx in the area indicated. Dashed vertical lines, sunset and sunrise; dotted lines, beginning and end of twilight.

The daily lynx activity showed a clear pattern, with the lowest activity during midday and high activity during twilight and night, irrespective of the duration of the day (Figs. 2 and 3). During spring and fall when days and nights were equally long, the activity patterns in all study areas were similar, with a period of low activity during the day and activity peaks during twilight evenings and twilight mornings. Activity at night was lower than at twilight but higher than during the day. In September, activity was similar during twilight evenings and twilight mornings; in March, activity was higher during twilight evenings, except for southern Norway, where activity did not differ. In the Bavaria–Czech Republic study area in June, activity peaked around sunrise and sunset. In southern Norway during midsummer, the twilight activity peaks were not pronounced (i.e. not very different from night-time levels), and the period of low activity during the day was longer. During polar day in northern Sweden and northern Norway, activity did not peak, but activity around noon was still lower than at midnight. In December in Bavaria–Czech Republic and southern Norway, a pronounced activity peak was observed only in the evening, but not in the morning. In northern Sweden and northern Norway during polar nights, lynx activity was still lowest around noon, but this period of low activity was short; during the rest of the day, activity was higher but without peaks after or before noon. Figs. 2 and 3 also show that the duration of the active phase of the activity cycle varied with the widening and narrowing of the photoperiod.

### Modelling approach

The model showed that the Eurasian lynx has a circadian activity pattern with low activity around noon and high activity at twilight and night. The highest activity was observed during twilight evenings, followed by twilight mornings and night (Table 3). We observed that activity varied significantly with moon phase, but this variation was very small. We did not find a significant interaction between moon phase and study area.

**Table 3:** Results of the linear mixed additive model with the response variable set to the percentage of time active per hour.

Variable	Estimate	p-value
Intercept	0.3354	
Day	0	
Night	0.0204	<0.001
Twilight evening	0.1404	
Twilight morning	0.1151	
Moon	−0.0002	0.005
Females	0	
Females with kittens	0.0209	<0.001
Male	0.0337	
Adult	0	0.011
Subadult	0.0349	
Autumn	0	
Spring	−0.0345	
Summer	−0.0003	<0.001
Winter	−0.0332	
Bavaria-Czech Republic	0	
Southern Norway	0.0123	
Northern Sweden	0.0242	0.129
Northern Norway	0.0308	
**R^2^ adj.**	**0.157**	

Also the status of the animal significantly influenced its activity. Male lynx were the most active, followed by females with kittens and then females without kittens. Generally, subadult lynx were significantly more active than adults. Also activity varied significantly with season (with autumn as the reference category) (Table 3). Lynx were more active in autumn and summer than in spring and winter. Lynx activity decreased from northern to southern latitudes (highest in northern Norway, lowest in Bavaria-Czech Republic).

**Figure 4:**
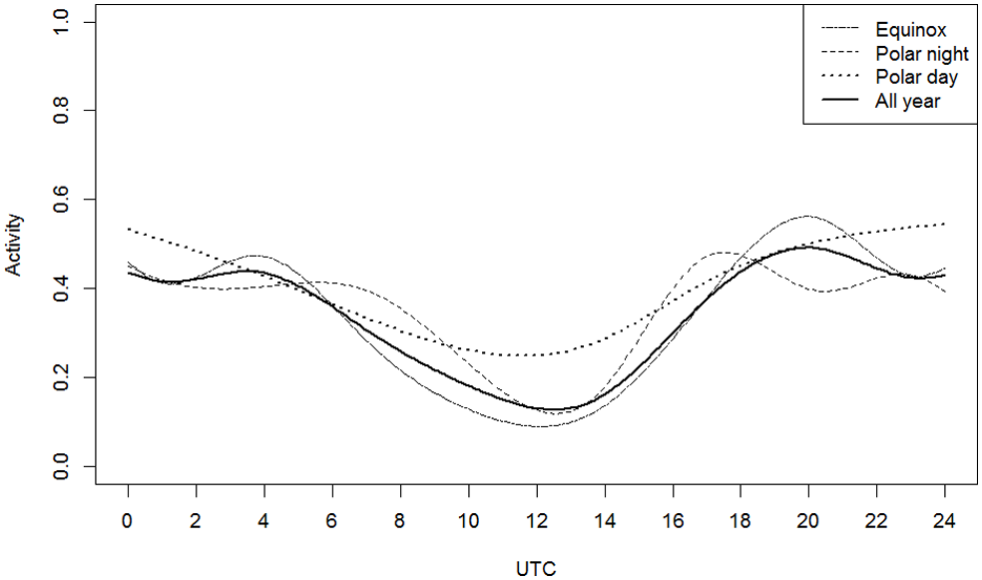
Lynx activity predicted by the linear mixed additive model for the response variable portion of time spent active per hour during one day (activity) a) throughout the year, and b) subsets of data representing different light conditions. Each covariable is predicted for the reference category adult, female, autumn, Bavaria–Czech Republic, and full moon ( = 0); therefore, only time influences the activity.

We included the time of day with an additive term (p-spline) in the model of the activity per hour and adjusted for the other covariates (Fig. 4). Activity was lowest at 12∶00 (noon) and thereafter increased steadily to the highest level at 20∶00 (twilight evening, early night). Activity remained high until early morning, with values>0.4 (lynx active 40% of the time). After morning, the activity level decreased to its minimum level at noon. The 95% confidence level for the predicted activity of these values and the following estimations were narrow; therefore, the 95% confidence intervals are not shown in the figures.

Season-specific models showed that even during polar night and polar day, activity around noon was low. However, the period of low activity was shorter during polar night and longer and less pronounced during polar day. Also during polar day, activity did not peak before or after the noon low. During equinox, activity peaked at twilight morning (4∶00) and at twilight evening (20∶00) (Fig. 4). The differences in the predicted activity patterns around equinox, polar night and polar day (Fig. 4) indicated an important influence of daylight duration on the lynx daily activity pattern.

**Figure 5:**
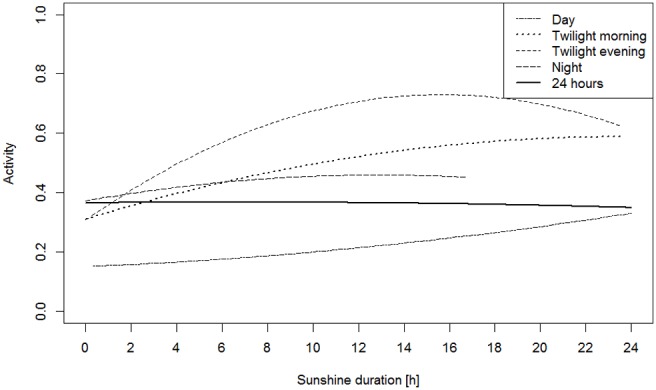
Lynx activity predicted by the linear mixed additive model for the response variable portion of time spent active (activity) for one 24–h period (whole day) and during the phases day, twilight morning, twilight evening and night depending on daylight duration. Each covariable is predicted for the reference category adult, female, autumn, Bavaria–Czech Republic, full moon ( = 0); therefore, only daylight influences the activity.

In our second analysis, we assessed the association between activity during the four day phases (twilight morning, day, twilight evening, and night) and daylight duration. The general 24-h activity level of the animals was not associated with the daylight duration (Fig. 5). The allocation of activity to the different day phases changed with respect to daylight duration (Fig. 5). The highest activity was during twilight evening, followed by twilight morning, night and day. Only when the daylight duration was very short did the night activity increase. With increasing daylight duration, activity at twilight evening strongly increased and reached a peak at 17 h of daylight and afterwards decreased again. Activity at twilight morning and during the day increased almost linearly with increasing daylight, whereas activity at night increased only slightly. The estimators for the other covariates are in line with those of the main model and are given in S3 Table. Model output of the linear mixed additive model for the response variable portion of active time for the whole day (24 h) and the phases day, twilight morning, twilight evening and night.

## Discussion

Our analysis of lynx activity patterns over a wide latitudinal range revealed as predicted that the overall daily activity level was not influenced by daylight duration. The distinct circadian pattern was even kept during polar night and polar day. However, the lynx activity pattern significantly varied with daylight duration. First, activity during twilight evening and twilight morning peaked on days with a full day–night cycle, as has been already shown in other studies [14], [15], while the amplitude of circadian variation in activity was lower during polar night and polar day. Second, the active phase ( =  alpha) was longer in summer than in winter. Contrary to our expectations, lynx activity varied significantly with moon phase, but this variation was very small. As predicted, subadult lynx were more active than adults. Among the adults, male lynx were more active than females, and females with kittens were more active than solitary females. Lynx were most active in autumn and summer. The general activity level in the study sites decreased from north to south.

We identified bimodal lynx activity patterns with lowest activity at midday and highest activity after sunset or before sunrise, although the variation of activity was lower in the polar environment. This finding for lynx in regions with polar nights and polar days contradicts the suggestions of van Oort et al. [25] for species living in polar environments. The polycyclic activity pattern of reindeer observed by these authors is probably reindeer specific and cannot be generalized for other species, or at least not for carnivores. This interpretation is in accordance with molecular evidence, which has shown that reindeer lack mechanisms for generating circadian rhythmicity [38], which seems to be a specific adaptation of this species. In contrast to reindeer, Eurasian lynx distribution is not limited to polar and boreal environments and covers a wide latitudinal range from northern Russia to northern India and the Mediterranean. Therefore, lynx physiology is adapted to a 24-h rhythm ruled by the day–night cycle. However, the general circadian pattern of lynx was adapted to the different light regimes. This could be seen in the smaller amplitude of activity during polar day and polar night than at equinox and a shorter period of the amplitude during polar day than polar night as well as lack of activity peaks at high latitudes during polar day and polar night.

Our results of lynx activity at lower latitudes and at times around the equinox support results of previous studies from Central Europe in areas with a complete day–night cycle [14], [15], [20]. In these areas, lynx activity is lowest during the day and highest at night, with peaks at twilight. This can be interpreted as the animals spending their days at a lair and hunting and feeding in the evening and night. Such a crepuscular activity pattern has also been shown for other felids, such as Canadian lynx (*Lynx canadensis*) [39], bobcat [40], Iberian lynx [41] and tiger (*Panthera tigris*) [42]. This pattern is probably strongly influenced by the activity patterns of their primary prey and the lynx hunting technique. Several studies have shown that red deer and roe deer, the principal prey of Eurasian lynx in the southern study regions, also have a clear crepuscular activity pattern [34], [43]–[46]. Lynx activity followed these crepuscular activity peaks, but was also high during the night, when deer are less active. One reason for this could be that darkness gives increased cover to the lynx, thereby impeding visual detection by the prey, which is crucial for a stalking predator. Their prey species rely more on olfactory or auditory information at night, whereas lynx, with their superior visual senses [11], might be more successful in catching prey during the night. Our results indicated that lynx activity patterns seem to follow that of its prey species. The lynx show pronounced bimodal activity peaks when the main prey species (red deer and roe deer) show bimodal activity during twilight and less pronounced activity peaks when the main prey species (reindeer) have a polycyclic activity pattern with no distinct peaks in activity at twilight [47]. Another reason for the crepuscular and night activity could be the avoidance of human activity, which has been shown to strongly influence circadian behaviour patterns of several carnivores [48]–[51]. This argumentation is supported by the fact that in all study regions, human persecution (either legal or illegal) is the main cause of lynx death, which might lead to the lynx behavioural adaption of avoiding periods when people are most active [52], [53]. On the other hand, lynx persecution by humans is a very young phenomenon from an evolutionary perspective, yet nocturnal activity of mammals is hypothesized to date back millions of years [17]. Therefore, human persecution might simply reinforce an innate characteristic and might not be the ultimate cause of the observed activity pattern. This interpretation is also supported by the finding that lynx activity was highest during twilight evening when human activity was also highest. If human disturbance was a major driver of lynx activity, one would in contrast expect highest lynx activity during twilight morning when human activity is lower.

The general 24-h activity level of the lynx was constant despite changes in daylight duration. This result shows that lynx do not have to be active longer, for example, to obtain enough food as the daylight duration increases. Consequently, it does not appear that hunting success is dependent on the day–night cycle because a lower hunting success would need to be compensated for by a higher hunting effort, which would lead to a higher activity level [54]. However, the timing of activity allocation changed with increasing daylight duration. The daytime activity level was lower than the activity level during all other day phases but increased with daylight duration. Despite this, night-time activity only slightly increased. Highest activity was at twilight evening, followed by twilight morning. Given the frequency of this pattern among carnivores, it seems that crepuscular and nocturnal activities are driven by optimal hunting conditions [55].

Theuerkauf et al. [10] argue that wolves can most easily detect prey under dim light conditions that occur on moonlight nights or at twilight. Although we identified lynx activity peaks at twilight, the moon phase did not have a meaningful influence on lynx activity, even though light conditions during nights with bright moon phases are similar to those at twilight. Therefore, light condition is probably not as important a factor as prey activity, as has been shown for some other carnivores, e.g. lions [56]. However, moonlight does not seem to influence the nocturnal activity levels of roe deer [45]. Accordingly, lynx have no advantage in increasing their activity at full moon, but would have an advantage at twilight, when roe deer are most active. Also puma activity does not vary with the level of moonlight, and jaguar activity even declines with increased moonlight [18]. On the other hand, African lions are less successful in obtaining prey on moonlit nights [27]. The effect of moonlight might also be masked by the weather. Clouds during full moon might result in the same light conditions as with no moon. We were not able to include local weather parameters in our study, but because of the large sample size, it should have been possible to detect a significant overall influence even without considering detailed weather parameters.

By studying lynx activity over a large geographic range, we tested whether lynx activity patterns already known for lynx in central Europe are characteristic across a wider part of their distribution. As previously shown, demographic differences in activity levels may be due to the energetic/physiological demands associated with social and reproductive behaviours. The home ranges of males of this polygamous species encompass the home ranges of several females [57], [58]. To patrol these large home ranges, males must cover larger distances than females, and this is reflected in the higher activity of males. Such a pattern has also been documented for male mountain lions [59]. In contrast to our results, Schmidt [14] found that the activities of female and male lynx were similar. They argued that although males have to allocate more time to patrolling their larger home range, females with kittens have to kill on average 1.5 times more deer per time unit [60]. We were also able to confirm that female lynx with kittens had a higher mean activity than females without kittens. At first glance, this result does not seem to follow expectations, as females with kittens stay longer in the den to lactate and warm the offspring. However, the denning period is actually short (6–8 weeks) and falls within the warmest period of the year (parturition end of May/beginning of June), and the females must intensively hunt afterwards because of their high energy needs during lactation. In addition, kittens are not able to follow their mother from kill to kill until late summer or autumn. Before this time, females have to return to the den after every hunt and for each feeding bout, which results in a much higher distance travelled. In addition, females with kittens have to hunt and kill more prey than females without kittens, which results in more activity [14], [22], [60]–[63]. In contrast, in northern Norway, females with kittens and females without kittens have the same kill rates, probably because of high densities of available prey [61]. The activity of female lynx with kittens increased during the day and in the morning; such a pattern has also been observed in previous studies of Eurasian lynx [14] [20] and for Canadian lynx [39].

Sub-adult lynx, which disperse between January to April [64], were more active across the 24-h period and during the day than adult individuals. The same behaviour was observed for Iberian lynx in their first year, although the diurnal activity decreased thereafter [4], and for African lions [65], and is believed to be a general pattern of nocturnal species [66]. Sub-adult animals have to leave their natal home range and search for areas not occupied by adult animals. In that time, they have to disperse over large distances, which might influence activity [64], [67]. Probably even more important is that the sub-adults are not experienced hunters and do not know where to find prey in unfamiliar areas. Therefore, it can be expected that they have to cover large distances and have a higher hunting effort, which increases overall activity levels. Higher activity during the day could be advantageous because the sub-adults might thereby minimize interactions with dominant territory holders, who are more active during twilight and at night [65] and who would aggressively drive the sub-adults out of their home range [68], [69].

Lynx were in general more active in autumn and summer than in spring and winter. We hypothesized that they would be more active in winter and during the mating season (February/March), when males move over longer distances to secure females for mating and when territorial marking is most intensive [70]. Also the kittens are larger in winter, requiring more prey to be supplied by the females. However, it is easier to hunt in winter because snow cover limits the mobility of their prey, while the movement of lynx is less affected [34], [45]. Moreover prey species are often clustered around feeding stations or on open ridges where snow is blown away [71]. At such sites, lynx can find prey without a high searching effort. Also meat decomposition is slower in winter, and it has been shown that the role of scavenging is not as important in that season [72]. During summer and autumn, prey is more dispersed, and the hunting effort is therefore probably higher. Also predation may include more neonates in summer and a higher loss of meat by invertebrate and vertebrate scavengers [72], [73], which would result in higher predation rates and a higher overall activity level. In addition, the observed seasonal differences can also depend on weather features, such as snow cover, which can hinder movements over large distances.

Lynx activity increased with latitude and was higher in northern study sites than in southern study sites. This difference could be related to a multitude of factors because the study site is a surrogate of different factors, such as various environmental conditions, different levels of human impact and different prey species. But it is likely that the observed differences are related to the size of the home ranges, which are 6 (males) to 8 (females) times larger in northern Norway (S2 Table Lynx density, hunting, prey species and light conditions of the study sites), and to the subsequent lower densities [74]. Therefore, the lynx have to travel longer distances to patrol their territory and to search for prey, forcing them to be more active [54].

The Eurasian lynx, with its large geographical distribution across Eurasia, shows a general circadian activity pattern with low activity at noon and high activity at night. Even during polar night and polar day, this general pattern is kept; however, it was modulated such that the differences between night-time and daytime activity were not as pronounced during polar night and the period with low activity was shorter during polar night.

Our results showed that the general activity level of the Eurasian lynx is the same regardless of light conditions and that only relative activity levels during different parts of the day and the distinctness of activity peaks change, being more or less pronounced. Therefore, we conclude that the lynx circadian, bimodal activity pattern is stable throughout the lynx range, even when polycyclic prey are hunted. According to historical records, the colonization of the northernmost range in Scandinavia was very recent, starting in the 1960s and reaching Finnmark (the northernmost study site) in the early 1990s [75]. Perhaps the observed activity patterns in the far north are only a relict characteristic with a genetic basis from lower latitudes, where activity was highly adaptive. These patterns might have been conserved in the subarctic biomes because of the short time interval and because maintaining these patterns incurs no cost. For the Eurasian lynx, light conditions do not appear to be the limiting factor for their hunting strategy as a stalking predator as they do not have to compensate for lack of darkness as cover and do not have to adapt to variable light conditions owing to moon phases. These points indicate that a crepuscular activity schedule provides an optimal hunting strategy, which enables a mid-sized carnivore to have a generalized diet and wide distribution.

In future research, other factors influencing Eurasian lynx activity have to be taken into consideration. First, the interaction of Eurasian lynx behaviour with fine temporal scale environmental conditions, such as temperature and snow, would provide information on how weather parameters influence the animals in different regions. This would form the basis for understanding how global change might influence the observed patterns. Second, as prey activity is an important driving factor of predator activity, simultaneous studies of predator and prey activities would be informative. Third, to evaluate anthropogenic factors, comparison of activity patterns of populations exposed to various levels of exploitation could reveal whether crepuscular and nocturnal activity patterns either are influenced by the environmental or have a genetic basis. Finally, we hope that this study will inspire other comparative analyses of activity data of other species, especially common prey species with similarly wide latitudinal distributions. The widespread use of GPS collars with activity sensors should allow the generation of vast amounts of untapped data that could be used to explore the relative impacts of a diversity of internal and external factors on animal chronobiology.

## Supporting Information

S1 Table General characteristics of the study sites.
(DOCX)

S2 Table Lynx density, hunting, prey species and light conditions of the study sites.
(DOCX)

S3 Table Model output of the linear mixed additive model for the response variable portion of active time for the whole day (24 h) and the phases day, twilight morning, twilight evening and night.
(DOCX)

S1 Dataset Data table, R-Code.
(ZIP)
