# Supplementary material for: Activity Patterns of Eurasian Lynx Are Modulated by Light Regime and Individual Traits over a Wide Latitudinal Range
Source: PLoS One. 2014 Dec 17;9(12):e114143. doi: 10.1371/journal.pone.0114143 (PMC4269461; doi:10.1371/journal.pone.0114143)
Supplement: S1 Table — General characteristics of the study sites. (DOCX) [file pone.0114143.s001.docx]

**General characteristics of the study sites**

| **Study site** | **Coordinates** | **Habitat characteristics** | **Elevation (m a.s.l.)** | **Institution** |
| --- | --- | --- | --- | --- |
| Bavaria/Czech Republic | 49°7'N, 13°36'E | Central European mixed mountainous forest dominated by Norway spruce (Pinus sylvestris), European beech (Fagus sylvatica) and white fir (Abies alba) in the central part, and cultivated land, mainly meadows, at the edge of the study site. | 650–1,450 | Bavarian Forest National Park and Šumava National Park |
| Southern Norway | 59°45'N, 9°40'E | Hilly area dominated by boreal forest dominated by Norway spruce and Scots pine (*Pinus sylvestris*) in valleys, and alpine tundra above the treeline | 0–1,500 | Norwegian Institute for Nature Research (NINA) |
| Northern Sweden | 67°00'N, 17°40'E | Mountainous area with valleys dominated by Scots pine, Norway spruce and mountain birch (*Betula pubescens*) at higher elevations and alpine tundra above the treeline. | 300–2,000 | Swedish University of Agricultural Sciences (SLU) |
| Northern  Norway | 70°00'N, 25°00'E | Area dominated by alpine tundra, followed by mountain birch forest and small patches of pine forest along the coast and in some lower valleys | 0–1,600 | Norwegian Institute for Nature Research (NINA) |
