# Supplementary material for: Activity Patterns of Eurasian Lynx Are Modulated by Light Regime and Individual Traits over a Wide Latitudinal Range
Source: PLoS One. 2014 Dec 17;9(12):e114143. doi: 10.1371/journal.pone.0114143 (PMC4269461; doi:10.1371/journal.pone.0114143)
Supplement: S2 Table — Lynx density, hunting, prey species and light conditions of the study sites. (DOCX) [file pone.0114143.s002.docx]

**Lynx density, hunting, prey species and light conditions of the study sites**

| **Study site** | **Lynx density per 100 km²** | **Mean home range size**  **MCP95 [km2] ^5^** | **Legal hunting** | **Main prey species** | **Light regime** |
| --- | --- | --- | --- | --- | --- |
| Bavaria/Czech Republic | 1.2 | m: 432 (N = 6)  f: 122 (N = 4) | no | Roe deer, red deer^1^ | Complete day–night cycle all year.  Longest day (June) 15.4 h; shortest day (December) 8 h |
| Southern Norway | 0.25 | m: 994 (N = 12)  f: 556 (N = 9) | yes | Roe deer, red deer, domestic sheep ^2,3^ | Complete day–night cycle except for May and June, which are without night (sun sets but light levels only drop to twilight levels in darkest period). Longest day (June) 18.3 h; shortest day (December) 5.6 h |
| Northern Sweden | 0.5 | m: 1660 (N = 4)  f: 874 (N = 8) | yes | Semi-domestic reindeer^4^ | Complete day-night-cycle only in spring and fall. 39 polar days in summer, 22 days in winter without daylight, just twilight |
| Northern Norway | 0.1 | m: 2360 (N = 6)  f: 870 (N = 17) | yes | Semi-domestic reindeer, domestic sheep^4^ | Complete day–night cycle only in spring and fall. 72 polar days in summer; 54 polar nights in winter |

^1^Podolski, I., Belotti E., Bufka L., Reulen H., Heurich M. (2013) Seasonal and daily activity patterns of free-living Eurasian lynx (*Lynx lynx*) in relation to availability of kills. Wildlife Biology 19(1) 69-77.

^2^Odden, J., Linnell, J.D.C. & Andersen, R. (2006) Diet of Eurasian lynx*, Lynx lynx*, in the boreal forest of southeastern Norway: the relative importance of livestock and hares at low roe deer density. European Journal of Wildlife Research 52, 237-244.

^3^Gervasi, V., Nilsen, E.B., Odden, J., Bouyer, Y. & Linnell, J.D.C. 2014. The spatio-temporal distribution of wild and domestic ungulates modulates lynx kill rates in a multi-use landscape. Journal of Zoology 292:175-183. DOI: 10.1111/jzo.12088.

^4^Mattisson, J., Odden, J., Nilsen, E. B., Linnell, J.D.C., Persson, J. & Andren, H. (2011) Factors affecting Eurasian lynx kill rates on semi-domestic reindeer in northern Scandinavia: Can ecological research contribute to the development of a fair compensation system? Biological Conservation 144, 3009-3017.

^5^ 95 % Minimum Convex Polygon
