# Supplementary material for: Activity Patterns of Eurasian Lynx Are Modulated by Light Regime and Individual Traits over a Wide Latitudinal Range
Source: PLoS One. 2014 Dec 17;9(12):e114143. doi: 10.1371/journal.pone.0114143 (PMC4269461; doi:10.1371/journal.pone.0114143)
Supplement: S3 Table — Model output of the linear mixed additive model for the response variable portion of active time for the whole day (24 h) and the phases day, twilight morning, twilight evening and night. (DOCX) [file pone.0114143.s003.docx]

**Model output of the linear mixed additive model for the response variable portion of active time for the whole day (24 h) and the phases day, twilight morning, twilight evening and night.**

|  | **24 h** | **Day** | **Twilight morning** | **Twilight evening** | **Night** |
| --- | --- | --- | --- | --- | --- |
|  | **Estimate** | **Estimate** | **Estimate** | **Estimate** | **Estimate** |
| **Intercept** | 0.366 | 0.151 | 0.311 | 0.308 | 0.372 |
| **Daylight duration** | 0.283 | 0.004 | 0.000 | 0.000 | 0.000 |
| **Daylight duration^2^** | −0.000 * | 0.000 *** | −0.000 *** | −0.002 *** | −0.001 ** |
| **Moon** | 0.000 | 0.000 | 0.001 | 0.001 | −0.001 |
| **Fem-kit** | 0.017** | 0.031* | −0.040* | −0.014 | −0.004** |
| **Male** | 0.036** | 0.020* | 0.037* | 0.031 | 0.062** |
| **Young** | 0.033** | 0.045** | 0.051 | 0.000 | 0.004 |
| **Spring** | −0.042*** | −0.001*** | −0.078*** | −0.122*** | −0.025*** |
| **Summer** | −0.006*** | 0.029*** | −0.016*** | −0.034*** | −0.003*** |
| **Winter** | −0.036*** | −0.006*** | −0.102*** | −0.036*** | −0.045*** |
| **N-Norway** | 0.041* | 0.017 | 0.053 | −0.033 | 0.079* |
| **S-Norway** | 0.019* | 0.014 | 0.006 | −0.068 | 0.023* |
| **N-Sweden** | 0.037* | 0.033 | 0.035 | −0.031 | 0.045* |
|  |  |  |  |  |  |
| **R2** | **0.124** | **0.244** | **0.136** | **0.185** | **0.085** |

Signif. codes: 0 ‘***’ 0.001 ‘**’ 0.01 ‘*’ 0.05 ‘.’ 0.1 ‘ ’ 1
